# Extraction and Physicochemical Characterization of Chitin from *Cicada* *orni* Sloughs of the South-Eastern French Mediterranean Basin

**DOI:** 10.3390/molecules25112543

**Published:** 2020-05-29

**Authors:** Aurelia Poerio, Chloé Petit, Jean-Philippe Jehl, Elmira Arab-Tehrany, João F. Mano, Franck Cleymand

**Affiliations:** 1 Jean Lamour Institute, University of Lorraine, UMR 7198 CNRS, 2 allée André Guinier-Campus Artem, BP 50840, F-54011 Nancy CEDEX, France; aurelia.poerio@univ-lorraine.fr (A.P.); chloe.petit9@etu.univ-lorraine.fr (C.P.); jean-philippe.jehl@univ-lorraine.fr (J.-P.J.); jmano@ua.pt (J.F.M.); 2 Laboratoire Ingénierie des Biomolécules, University of Lorraine, TSA 40602, F-54518 Vandoeuvre-lès-Nancy, France; elmira.arab-tehrany@univ-lorraine.fr; 3 Department of Chemistry, CICECO—Aveiro Institute of Materials, University of Aveiro, 3810-193 Aveiro, Portugal

**Keywords:** *Cicadaorni* sloughs, chitin, extraction, physicochemical characterization

## Abstract

Chitin is a structural polysaccharide of the cell walls of fungi and exoskeletons of insects and crustaceans. In this study, chitin was extracted, for the first time in our knowledge, from the *Cicada* *orni* sloughs of the south-eastern French Mediterranean basin by treatment with 1 M HCl for demineralization, 1 M NaOH for deproteinization, and 1% NaClO for decolorization. The different steps of extraction were investigated by Fourier Transform Infrared Spectroscopy (FTIR), X-ray Diffraction (XRD), Thermogravimetric Analysis (TGA), and Scanning Electron Microscopy (SEM). Results demonstrated that the extraction process was efficiently performed and that *Cicada* *orni* sloughs of the south-eastern French Mediterranean basin have a high content of chitin (42.8%) in the α-form with a high degree of acetylation of 96% ± 3.4%. These results make *Cicada* *orni* of the south-eastern French Mediterranean basin a new and promising source of chitin. Furthermore, we showed that each step of the extraction present specific characteristics (for example FTIR and XRD spectra and, consequently, distinct absorbance peaks and values of crystallinity as well as defined values of maximum degradation temperatures identifiable by TGA analysis) that could be used to verify the effectiveness of the treatments, and could be favorably compared with other natural chitin sources.

## 1. Introduction

Chitin is a copolymer of *N*-acetyl-d-glucosamine and d-glucosamine units linked with β-(1–4) glycosidic bond, composed predominantly of *N*-acetyl-d-glucosamine units and represents the second most abundant polysaccharide after cellulose [1]. Due to their excellent properties, including biocompatibility, nontoxicity [2], and biodegradability [3], chitin and its derivatives, such as chitosan (deacetylated form of chitin), have recently become of great interest for medical and pharmaceutical applications [4,5]. Even though chitosan is mostly used for biomedical applications due to its higher solubility in water and other traditional solvents such as acetic acid [6], chitin can be solubilized in some other solvents, such as ionic liquids [7,8]. Due to the versatile properties of the material, it can be used to fabricate scaffolds [3], hydrogels [9], or microspheres [10], showing promising physiochemical, mechanical, and biological properties for different applications, such as in the biomedical field for wound healing [11], vascularization [12], and bone repair [7]. Chitin represents the primary structural component of cell walls in fungi and of the exoskeletons of arthropods, such as crustaceans and insects [1]. Some species that belong to these subphyla are periodically forced to replace their old cuticle with a new one in a process called ecdysis. The lost cuticle is mainly made of chitin, but also of proteins and calcium carbonate, creating a rigid structure with the role of protecting the inner soft tissues from injury [13]. Therefore, these cuticles can represent a source of chitin. Depending on the source, the chitin can exist in three different forms: α-, β-, and γ-chitin that present an antiparallel, parallel, and alternated arrangements of polymer chains, respectively [14]. α-chitin is mostly found in fungi [15], arthropod exoskeleton, and shells of crustaceans [1], β-chitin is mostly found in squid pens [16], and γ-chitin is mostly found in the beetle family Lucanidae [17]. Depending on the source and the extraction conditions, the obtained chitin can have different degree of acetylation (DA), molecular weight (M_w_) and crystallinity index (CrI), which influence its physicochemical and biological properties and therefore its applications [18]. Several methods have been developed to extract chitin from different natural sources, generally following a common protocol: the elimination of minerals (demineralization), proteins (deproteinization), and pigments (decolorization) until the final product: the chitin. These extraction methods can have either a biological or chemical nature [19]. Biological extraction methods are based on the use of lactic acid-producing bacteria for the demineralization, thanks to the reaction of lactic acid with calcium carbonate and the subsequent formation of calcium lactate that can be removed by precipitation, while the deproteinization is mainly based on the use of proteolytic bacteria, which produce proteases that eliminate proteins [20]. Interestingly, some bacteria have shown the advantage of taking part in both the deproteinization and demineralization processes [21]. Chemical extraction methods are the most used in both industrial and laboratory production and involve an acid treatment, mostly with hydrochloric acid (HCl) ranging from 0.5 to 2 M, to dissolve the calcium carbonate (demineralization), followed by alkaline treatment with sodium hydroxide (NaOH) to dissolve proteins (deproteinization) [22]. A recent alternative to the classic chemical extraction method is microwave-assisted extraction, which is based on the use of a different energy source to accomplish the same processes of demineralization, deproteinization, and decolorization, allowing enhanced reaction rates [23], but less information is available in the literature. Although chemical treatments can affect the physiochemical properties of the extracted chitin more than the biological ones, the short time of processing compared to the biological methods makes them the most commonly used treatments [18]. In industrial processing, chitin is mainly extracted from crustaceans (crab and shrimp shells), but different studies have demonstrated that chitin extracted from different sources has distinct characteristics [24]. For example, the chitin extracted from the cuticle of insects has lower levels of inorganic material compared to crustacean shells, making the demineralization process and the subsequent deacetylation process easier [25]. Even lower levels of inorganic materials are present in chitin extracted from fungal sources, making, in some cases, the demineralization process unnecessary [26,27]. Differences can also be present between the two sexes of the same species, such as in Grasshopper, where the chitin content is higher in males than female [28]. For this reason, it is important to characterize the chitin extracted from new sources. Furthermore, because of the high costs and increased demand, new sources of chitin are needed [29]. Cicadas are insects that belong to the order of Hemiptera and to the superfamily of Cicadoidea that lives in temperate climates [30]. *Cicada orni* represents the most abundant species of cicada in France (representing the 27% of all cicada species identified in the country), and especially in the south of France [31]. Despite the absence of updated information, there is no evidence of a decrease in the abundance of cicadas in the south of France [32]. The annual presence of *Cicada orni* could allow us to expect similar quantities of chitin every year. Their sloughs represent an important source of chitin. Extraction of chitin from cicada has been reported by a limited number of studies [30,33,34,35,36,37]. The aim of this study was not only the extraction and physicochemical characterization of the chitin obtained from *Cicada orni*, but, for the first time, we propose a detailed analysis of all the steps of the extraction process. In this study, we used a slightly modified version of Luo et al.’s extraction protocol [33]. The physiochemical properties of chitin were characterized by Fourier transform infrared spectroscopy (FTIR), X-ray diffraction (XRD), thermogravimetric analysis (TGA), and scanning electron microscopy (SEM).

## 2. Results and Discussion

### 2.1. Chitin Extraction

**Figure 1:**
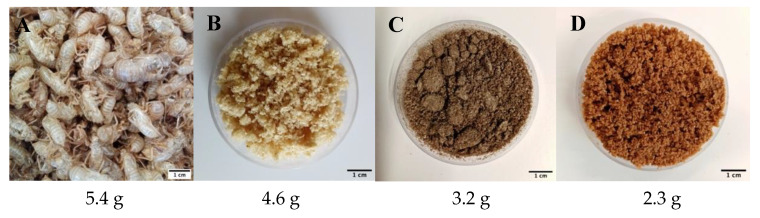
Schematic illustration of the raw material and all the other samples after the extraction steps. The weight of the raw material (**A**) after washing and drying it in the oven at 50 °C for 24 h was 5.4 g. The weight of dried demineralized (**B**), deproteinized (**C**), and decolorized samples (chitin-**D**) was 4.6, 3.2, and 2.3 g, respectively.

Figure 1 shows pictures of the samples corresponding to all the steps of chitin extraction with their relative weights after each step. Raw material corresponds to cicada sloughs washed and dried at 50 °C for 24 h and represents the initial quantity (5.4 g). The decolorized samples correspond to the final product chitin. After each step, the powder was dried and weighed, and the difference of weight before and after the demineralization and deproteinization processes was used to calculate, as described in Equation (2), the percentage content of minerals and proteins present in the raw material. The mineral and protein contents were estimated to be 14.8% and 30.4%, respectively. The chitin content (%) of *Cicada orni* sloughs of the south-eastern French Mediterranean basin studied, defined in the following as *Cicada orni* (CO), and calculated using the Equation (1), was 42.6%. The chitin, protein, and mineral content of *Cicada orni* sloughs are similar to the only reported study on chitin extraction from cicada sloughs. According to Sajomsang and Gonil [35] cicada sloughs consist of approximately 37% chitin, 40% proteins, and 12% minerals. Only one other study reported the chitin content of cicada but, this time, from the whole body of six cicada species collected from the Mediterranean region of Turkey. The chitin content was in the range of 4.97–8.84% [30]. Table 1 summarizes some of the chitin content from different sources. The chitin content obtained in this study is higher compared to other sources, (Table 1), including seafood, which represent the primary sources of chitin. According to these results, extracting chitin from cicada sloughs offers the advantage of the production of higher quantities of final product for a given initial source biomass. However, a disadvantage of this source of chitin is that its availability is limited to a few months of the year. Comparable chitin contents have been reported for squid gladius but, in that organism, the chitin is type β [38].

### 2.2. FTIR Analysis

Since chitin is a copolymer of *N*-acetyl-d-glucosamine and d-glucosamine, DA is an important factor used to measure the quality of chitin, being obtained from the ratio of acetylated group (*N*-acetyl-d-glucosamine) to deacetylated (d-glucosamine) amino groups [15]. FTIR spectra were used to (1) to analyze the demineralization, deproteinization, and decolorization processes, (2) to characterize the extracted chitin, and (3) to calculate the degree of acetylation.

**Figure 2:**
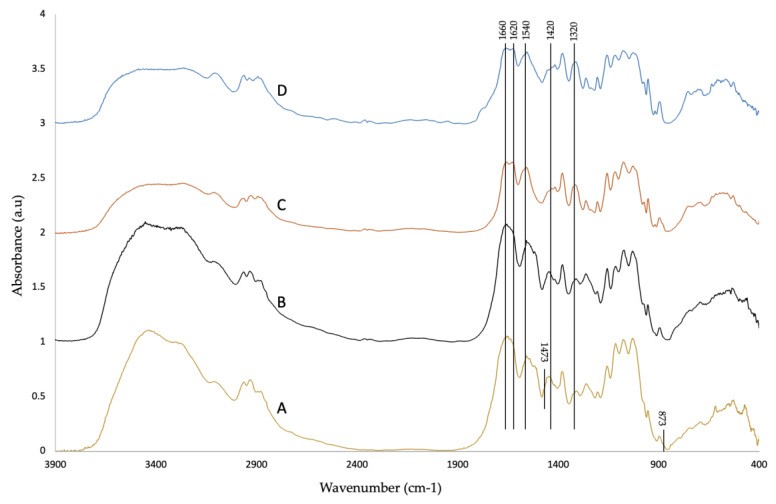
FTIR spectra of (**A**) raw material, (**B**) demineralized, (**C**) deproteinized, and (**D**) decolorized samples.

Figure 2 shows the FTIR spectra of raw material, demineralized, deproteinized, and decolorized samples. The trend of the absorbance of all samples is similar to the typical trend of chitin [52]. There is no remarkable difference between raw material and demineralized sample spectra. According to Gbenebor, [53], virgin crab and shrimp shell spectra present typical peaks for CaCO_3_ at 1473 and 874 cm^−1^ and 1443 and 874 cm^−1^, respectively, and these peaks reduced in intensity after demineralization with 1 M HCl. Raw material spectra present only small peaks at these wavelengths. The absence of these peaks might be due to the lower mineral content cicada sloughs (12–14%) [35] compared with shrimp (30–40%) [34,48] and crab shells (30.3–44%) [14,48]. Instead, there are differences between the demineralized and deproteinized samples. According to Hassainia, α-chitin presents two absorption peaks at about 1660 and 1627 cm^−1^ (due to the C=O stretching vibration secondary amide stretch of the amide I), while β-chitin presents only one band at 1656 cm^−1^ [15]. After deproteinization, the presence of two distinct peaks at about 1650 and 1621 cm^-1^ confirms α-chitin structure. These peaks are attributed to vibration modes of amide I, so their resolution in two separate peaks is probably due to the elimination of proteins [54]. In fact, only one peak is present in the raw material and demineralized sample. Deproteinized and decolorized samples present the same peaks, proving that pigments cannot be identified from these spectra. The degree of acetylation (DA) was calculated using the Brugnerotto expression (Equation (4)), which was the most reliable method in previous studies and in agreement with H-NMR and C-NMR data [55]. The peak at 1320 cm^−1^ is specific of *N*-acetyl glucosamine and it is used for the determination of the DA with the peak at 1420 cm^−1^ [52]. DA was calculated using the average of the absorption values at 1420 and 1320 cm^−1^ (see Figure S1 in Supplementary Materials for details about the baselines used to calculate the DA). The resulting DA was 96.3 ± 3.4. Table 2 summarizes the DA and crystallinity index (CrI) (that will be discussed below) of chitin from different sources. The obtained DA was similar to that obtained by Sajomsang and Gonil from cicada sloughs [35] and comparable to that reported in the literature from different sources (Table 2). The DA is the parameter that affects the solubility of chitin and its derivatives the most. In fact, when the DA is below 50%, the product is already called chitosan, and its solubility in acidic solutions increases with the reduction of the DA [56].

### 2.3. X-ray Diffraction

**Figure 3:**
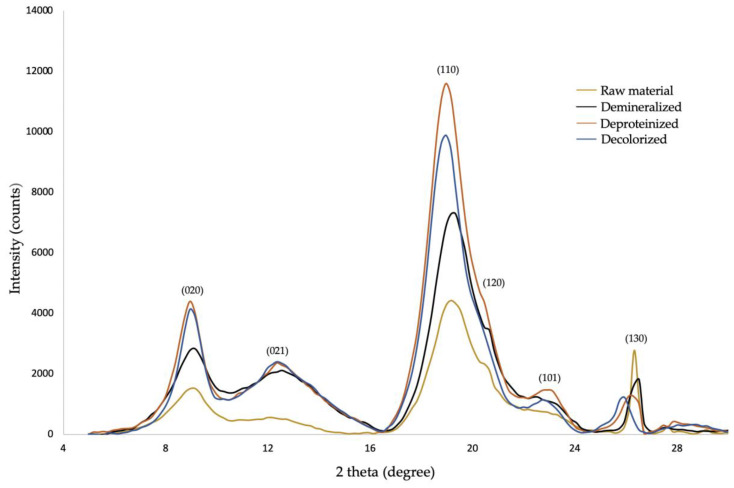
X-ray diffractograms of raw material, demineralized, deproteinized, and decolorized samples.

In nature, chitin is arranged in crystalline microfibrils [60]. The crystalline structure can be analyzed by X-ray diffraction (XRD). Figure 3 shows the XRD patterns of the demineralized, deproteinized, and decolorized steps of chitin extraction. All samples present six peaks at about 9.3°, 12.7°, 19.5°, 20.8°, 23.4°, and 26.3° that correspond to the six typical crystalline planes (020), (021), (110), (120), (101), and (130), respectively, except for the peak at 20.8° (120), which was decreased in the deproteinized sample and absent in the decolorized one. The positions and the amplitude of these peaks are in good agreement with those reported for five other sources of chitin, including cicada sloughs [33]. According to Jang [17], α-chitin has four crystalline reflections at 9.6, 19.6, 21.1, and 23.7, while β-chitin has two crystalline reflections at 9.1 and 20.3. *Cicada orni* results indicate that the extracted chitin is the α structure. Crystallinity index (CrI) is an important characteristic influencing the physical and biological properties of chitin, including solubility and biodegradability [59]. In particular, the higher the degree of crystallinity, the lower the solubility [60] and, accordingly, the biodegradability [61]. CrI was calculated using Equation (5). Results show that CrI was 68.6% for the raw material, 55.8% for the demineralized sample, 73.4% for the deproteinized sample, and 72.1% for the decolorized sample. Our hypothesis is that the removal of minerals may induce a diminution of CrI because of the absence of calcium carbonate crystals. After deproteinization, the increase of CrI may be due to a rearrangement of polymer chains after the removal of proteins. The CrI of chitin extracted from CO (72.1%) is within this range of chitin extracted in the literature from different sources, between 57.8% and 89.7% (Table 2).

### 2.4. Thermogravimetric Analysis

**Figure 4:**
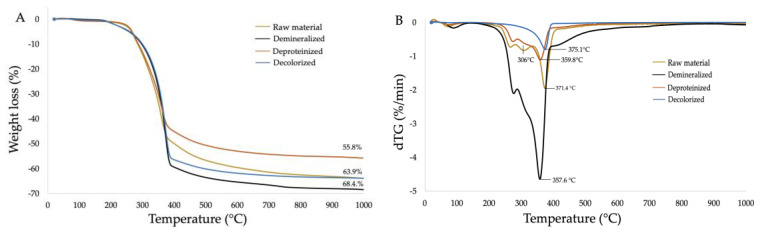
(**A**) TGA and (**B**) DTG of raw material, demineralized, deproteinized, and decolorized samples.

Thermogravimetric analysis (TGA), a measure of the change in mass of a substance as a function of temperature, is another important factor that defines the possible application of chitin [62] and helps to identify the type of chitin. TGA of raw material, demineralized, deproteinized, and decolorized samples is shown in Figure 4A. The maximum weight loss for all samples was obtained between 200 and 400 °C, and was 63.9% for the raw material, 68.4% for the demineralized sample, 55.8% for the deproteinized sample, and 63.9% for the decolorized sample. The maximum weight loss obtained from chitin extracted from cicada sloughs by Sajomsang and Gonil [37] was 66.4%, while the maximum weight loss obtained from chitin extracted from the whole body of cicadas was in the range of 72.2–88.3% [30]. Figure 4B shows the derivative thermogravimetric analysis (DTG) of the four samples. The thermal degradation of raw material occurs in at least four different degradation steps, that of demineralized and deproteinized samples in three steps, and that of decolorized samples in two steps. The temperature at which the maximum degradation of the samples occurred was 371.4, 357.6, 359.8, and 375.1 °C for raw material, the demineralized, deproteinized, and decolorized specimen steps, respectively. According to the literature, the maximum distortion temperature of α-chitin is usually higher than 350 °C, while the value of for β-chitin is usually lower than 350 °C [16]. The maximum degradation temperature of chitin from CO was 375.1 °C, confirming that it is α-chitin. This result is similar to the maximum degradation temperature of chitin extracted from cicada sloughs described by Sajomsang and Gonil [35], which was 362 °C, while for chitin extracted from the whole body of cicadas, it was in the range of 339.9–412.7 °C [30]. All samples presented a peak around 100 °C corresponding to water evaporation. The peak around 306 °C in raw material decreased in the demineralized sample and disappeared in deproteinized and decolorized samples. This suggests that it could be related to the demineralization process.

### 2.5. Scanning Electron Microscopy

**Figure 5:**
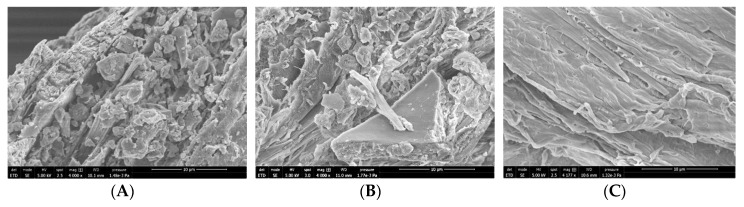
SEM images of (**A**) raw material, (**B**) demineralized, and (**C**) decolorized samples.

Figure 5 shows scanning electron microscopy (SEM) images of the surface of the raw material (Figure 5A), and demineralized (Figure 5B) and decolorized (Figure 5C) specimens. Raw material surface exhibits a rough morphology. We hypothesize that chitin has a lamellar organization intercalated with some granular material (that could correspond to minerals and proteins) that seems to decrease in demineralized and in decolorized samples, where only the flat lamellar structure seems to remain. This decrease could demonstrate the effective removal of minerals and proteins, respectively [35].

## 3. Materials and Methods

### 3.1. Extraction of Chitin from Cicada orni

*Cicada orni* sloughs were taken in August 2017 in the south-eastern French Mediterranean basin, especially in La Seyne sur Mer in a place called PinRolland. Chitin was extracted with a modified protocol based on Luo et al.’s work [33]. *Cicada orni* sloughs were washed with distilled water and dried in an oven at a temperature of 50 °C for 24 h. Then they were mechanically ground in a mortar. Specimen were demineralized with 1 M HCl aqueous solution (1:15 ratio of solid sample to solution) in a 30 °C water bath at 200 rpm for 2 h to remove calcium carbonate and other calcium salts. Then they were filtered out and washed with deionized water until neutral pH was detected. The reaction of demineralization is the following [63]:

CaCO_3_ + 2HCl → CaCl_2_ + H_2_O + CO_2_ (1)

In more detail, HCl reacts with calcium carbonate to produce an aqueous solution of calcium chloride (CaCl_2_) and CO_2_. Specimens were dried in the oven at 50 °C for 24 h and weighed to calculate the amount on minerals present in *Cicada orni* sloughs. For deproteinization, a treatment with 1 M NaOH solution (1:15 ratio of solid sample to solution) was done, and then refluxed at 90 °C for 2 h to remove proteins. The specimen was also dried in the oven at 50 °C for 24 h and weighed to calculate the amount of protein present in *Cicada orni* sloughs. Insoluble material was filtered and washed extensively with deionized water until neutral pH. For the decolorization, the sample was treated with 1% sodium hypochlorite (NaClO) (1:30 ratio of solid sample to solution) for 30 min at 25 °C room temperature, filtered, and washed with distilled water, and treated another two times with 1% NaClO for 10 min, until the water was transparent. After this last step, specimens were washed extensively to remove any residual chemicals.

### 3.2. Chitin Content

Chitin content was calculated by measuring the weight of samples before and after the extraction (always dried in an oven at 60 °C overnight). The chitin content (%) was calculated using the following formula:

(2) $$\mathrm{Chitin}\;\mathrm{content}\;\left(\%\right)=\frac{\mathrm{Dried}\;\mathrm{chitin}\;\mathrm{extracted}\;\left(g\right)}{\mathrm{Raw}\;\mathrm{material}\;\left(g\right)}\times 100$$

The mineral content (%) of cicada sloughs was calculated using the following formula:

(3) $$\mathrm{Mineral}\;\mathrm{content}\;\left(\%\right)=\frac{\left(W1-W2\right)}{W1}\times 100$$

where W1 is the raw material and W2 is the weight of the dried sample after demineralization. The protein content was calculated using the same formula, attributing the weight of the dried demineralized sample to W1 and the weight of the dried deproteinized sample to W2. The weight of the remaining material after the decolorization process represents the dried chitin extracted.

### 3.3. FTIR Analysis 

Dried powder of raw material, demineralized, deproteinized, and decolorized *Cicada orni* samples were used for FTIR analysis. Spectra were collected in a range between 4000 to 400 cm^−1^ with a Nicolet 6700 Spectrophotometer by accumulation of 64 scans with a resolution of 4 cm^−1^ in KBr pellets (1 mg of the sample in 100 mg of KBr). The degree of acetylation (DA) was calculated from the Absorbance (A) ratios according to the Brugnerotto method [52]:

(4) $$\mathrm{DA}\;\left(\%\right)=\frac{\frac{A1320}{A1420}-0.3822}{0.03133}\times 100$$

where the values of the absorbance at 1420 cm^−1^ and 1320 cm^−1^ were calculated using the baselines shown in Figure S1. The DA was expressed as average of results from eight FTIR spectra.

### 3.4. X-ray Diffraction 

Powder X-ray diffraction (XRD) (D8 Advance, Bruker, Billerica, MA, USA) analysis of demineralized, deproteinized, and decolorized steps were record using a D8 Advance X-ray diffractometer. Data were collected at a scan rate of 1°/min with the scan angle from 5–40°. The crystallinity indexes (CrI) were calculated using the following equation [57]:

(5) $$\mathrm{CrI}\;\left(\%\right)=\frac{I_{110}-I_{\mathrm{am}}}{I_{110}}\times 100$$

where I_110_ is the maximum intensity of the crystalline region at 20° and I_am_ is the maximum intensity of amorphous diffraction at 16°.

### 3.5. Thermogravimetric Analysis 

The thermal degradation properties of demineralized, deproteinized, and decolorized powdered specimens were analyzed using a thermogravimetric analyzer equipped of a Setaram microbalance ((Setsys Ev. 1750, Setaram, Kep Technologies, Lyon, France). The samples (10 mg) were heated from 20 °C to 1000 °C at a constant heating rate of 10 °C/min under argon atmosphere.

### 3.6. Scanning Electron Microscopy 

The surface morphologies of the samples were observed using a Quanta 650-FEG (FEI, Hillsboro, OR, USA) scanning electron microscope (SEM), model, manufacturer, city, country. The samples (raw material, demineralized, deproteinized, and decolorized) were dried, fixed on an adhesive tape, and coated with a gold layer. The images were taken at an acceleration voltage of 5 kV and with a magnification of 10 μm.

## 4. Conclusions

In this study, we extracted chitin from *Cicada orni* sloughs of the south-eastern French Mediterranean basin using a chemical method. We obtained a chitin yield of 42.6%, which is higher than that of other studies. FTIR, DRX, and TGA analysis confirmed that the chitin extracted from *Cicada orni* is in the α-form, with a DA of 99.6% ± 3.4%, a CrI of 72.1%, and a maximum degradation temperature of 375.1 °C. *Cicada orni* sloughs of the south-eastern French Mediterranean basin show great potential as an ecological alternative source of chitin. Furthermore, for the first time, we analyzed and described all the steps of the extraction process to compare the effectiveness of the treatments and different extraction conditions. For example, FTIR spectra showed the formation of two distinct peaks at 1650 and 1621 cm^−1^ after the deproteinization step that seem to be due to the elimination of proteins or, at least, to their decrease.

## Figures and Tables

**Table 1:** Chitin content from different sources.

Source	Chitin Content %	Ref.
*Cicada orni* sloughs (this study)	42.6	
Mushrooms	5.9–7.4; 4.31–9.66; 1.87–6.93	[15,39,40]
Grasshopper	4.71–11.84	[28]
Whole cicada body	4.97–8.84	[30]
Cicada sloughs	37	[35]
Squid gladius ^1^	40–42; 31.27; 31	[37,41,42]
House cricket	4.3–7.1	[43]
Worm of giant flour	4.77–5.43	[44]
Colorado potato beetles	20	[45]
Beetle (*H. parallela*)	15	[44]
Shrimp wastes	10.13; 3.12–17.36; 7.2	[46,47,48]
Crab shells	27.4; 17.35–20.62	[48,49]
Brine shrimp cysts	29.3–34.5	[50]
Hornet (*Vespa velutina*)	11.7	[51]

^1^ Chitin extracted from squid gladius is β-chitin.

**Table 2:** Degree of acetylation (DA) and crystallinity index (CrI) of chitin from different sources.

Source	DA %	CrI %	Ref.
*Cicada orni* sloughs (this study)	96.3 ± 3.4	72.1	
Mushrooms	63.4–69.8	63	[15]
Grasshopper	108.5–180.7	75–80	[28]
Cicada sloughs	90.8–102.3	89.7	[35]
Squid gladius ^1^	90.7–101.2; 96	72.3–85; 74.9	[38,42]
House cricket	108.1	88.02	[43]
Worm of giant flour	82.39–101.39	67.82–57.62	[44]
Beetle	93.1	89.05	[57]
Crab	78.5	67.8	[48]
Shrimp	88.5; 96; 65.6–99.4	64.1; 88; 79.4–87.4	[48,58,59]
Hornet (*Vespa velutina*)	95.44		[51]

^1^ Chitin extracted from squid gladius is β-chitin.

## Supplementary Materials

The following are available online, Figure S1 Representation of the baselines used to calculate the DA.

## References

[1] Rinaudo M. (2006). Chitin and chitosan: Properties and applications. Prog. Polym. Sci..

[2] Rejinold N.S., Nair A., Sabitha M., Chennazhi K.P., Tamura H., Nair S.V., Jayakumar R. (2012). Synthesis, characterization and in vitro cytocompatibility studies of chitin nanogels for biomedical applications. Carbohydr. Polym..

[3] Onishi H., Machida Y. (1999). Biodegradation and distribution of water-soluble chitosan in mice. Biomaterials.

[4] Park B.K., Kim M.M. (2010). Applications of Chitin and Its Derivatives in Biological Medicine. Int. J. Mol. Sci..

[5] Alves N.M., Mano J.F. (2008). Chitosan derivatives obtained by chemical modifications for biomedical and environmental applications. Int. J. Biolog. Macromol..

[6] Shamshina J.L. (2019). Chitin in ionic liquids: Historical insights into the polymer’s dissolution and isolation. A review. Green Chem..

[7] Silva S.S., Duarte A.R.C., Oliveira J.M., Mano J.F., Reis R.L. (2013). Alternative methodology for chitin-hydroxyapatite composites using ionic liquids and supercritical fluid technology. J. Bioact. Compat. Polym..

[8] Tolesa L.D., Gupta B.S., Lee M.J. (2019). Chitin and chitosan production from shrimp shells using ammonium-based ionic liquids. Int. J. Biolog. Macromol..

[9] Nagahama H., Nwe N., Jayakumar R., Koiwa S., Furuike T., Tamura H. (2008). Novel biodegradable chitin membranes for tissue engineering applications. Carbohydr. Polym..

[10] Wang Y., Li J., Li B. (2017). Chitin microspheres: A fascinating material with high loading capacity of anthocyanins for colon specific delivery. Food Hydrocoll..

[11] Singh R., Chacharkar M.P., Mathur A.K. (2008). Chitin membrane for wound dressing application-preparation, characterisation and toxicological evaluation. Int. Wound J..

[12] Sağlam E.İ., Kutlu İ.C., Haberal O.E., Yüksekkaya M., Kılıçarslan Ö., Güran Ş. (2019). Chitin increases the angiogenesis in chorioallantoic membrane model in the presence of testosterone and progesterone. Gulhane Med. J..

[13] Merzendorfer H. (2003). Chitin metabolism in insects: Structure, function and regulation of chitin synthases and chitinases. J. Exp. Biol..

[14] El Knidri H., Belaabed R., Addaou A., Laajeb A., Lahsini A. (2018). Extraction, chemical modification and characterization of chitin and chitosan. Int. J. Biolog. Macromol..

[15] Hassainia A., Satha H., Boufi S. (2018). Chitin from Agaricus bisporus: Extraction and characterization. Int. J. Biolog. Macromol..

[16] Wu Q., Jungstedt E., Šoltésová M., Mushi N.E., Berglund L.A. (2019). High strength nanostructured films based on well-preserved β-chitin nanofibrils. Nanoscale.

[17] Jang M.-K., Kong B.-G., Jeong Y.-I., Lee C.H., Nah J.-W. (2004). Physicochemical characterization of α-chitin, β-chitin, and γ-chitin separated from natural resources. J. Polym. Sci. A Polym. Chem..

[18] Kumirska J., Weinhold M.X., Thöming J., Stepnowski P. (2011). Biomedical Activity of Chitin/Chitosan Based Materials—Influence of Physicochemical Properties Apart from Molecular Weight and Degree of N-Acetylation. Polymers.

[19] Pighinelli L. (2019). Methods of Chitin Production a Short Review. AJBSR.

[20] Arbia W., Arbia L., Adour L., Amrane A. (2013). Chitin Extraction from Crustacean Shells Using Biological Methods—A Review. Food Technol. Biotechnol..

[21] Sorokulova I., Krumnow A., Globa L., Vodyanoy V. (2009). Efficient decomposition of shrimp shell waste using Bacillus cereus and Exiguobacterium acetylicum. J. Ind. Microbiol. Biotechnol..

[22] Hamed I., Özogul F., Regenstein J.M. (2016). Industrial applications of crustacean by-products (chitin, chitosan, and chitooligosaccharides): A review. Trend. Food Sci. Technol..

[23] El Knidri H., Dahmani J., Addaou A., Laajeb A., Lahsini A. (2019). Rapid and efficient extraction of chitin and chitosan for scale-up production: Effect of process parameters on deacetylation degree and molecular weight. Int. J. Biolog. Macromol..

[24] Rameshthangam P., Solairaj D., Arunachalam G., Ramasamy P. (2020). Chitin and Chitinases: Biomedical and Environmental Applications of Chitin and its Derivatives. JEN.

[25] Badawy R.M., Mohamed H.I. (2015). Chitin extraction, Composition of Different Six Insect Species and Their Comparable Characteristics with That of the Shrimp. J. Am. Sci..

[26] Jones M., Kujundzic M., John S., Bismarck A. (2020). Crab vs. Mushroom: A Review of Crustacean and Fungal Chitin in Wound Treatment. Mar. Drug..

[27] Abo Elsoud M.M., El Kady E.M. (2019). Current trends in fungal biosynthesis of chitin and chitosan. Bull. Natl. Res. Cent..

[28] Kaya M., Lelešius E., Nagrockaitė R., Sargin I., Arslan G., Mol A., Baran T., Can E., Bitim B. (2015). Differentiations of Chitin Content and Surface Morphologies of Chitins Extracted from Male and Female Grasshopper Species. PLoS ONE.

[29] Dutta P.K., Dutta J., Tripathi V.S. (2004). Chitin and chitosan: Chemistry, properties and applications. J. Sci. Ind. Res..

[30] Mol A., Kaya M., Mujtaba M., Akyuz B. (2018). Extraction of high thermally stable and nanofibrous chitin from *Cicada* (Cicadoidea). Entomol. Res..

[31] Puisasant S. (2006). Contribution à la Connaissance des Cigales de France: Geéonemie et Écologie des Populations (Hemiptera, Cicadidae).

[32] Blondel J., Aronson J. (1999). Biology and Wildlife of the Mediterranean Region.

[33] Luo Q., Wang Y., Han Q., Ji L., Zhang H., Fei Z., Wang Y. (2019). Comparison of the physicochemical, rheological, and morphologic properties of chitosan from four insects. Carbohydr. Polym..

[34] Bertuzzi D.L., Becher T.B., Capreti N.M.R., Amorim J., Jurberg I.D., Megiatto J.D., Ornelas C. (2018). General Protocol to Obtain D-Glucosamine from Biomass Residues: Shrimp Shells, Cicada Sloughs and Cockroaches. Glob. Chall..

[35] Sajomsang W., Gonil P. (2010). Preparation and characterization of α-chitin from cicada sloughs. Mater. Sci. Eng. C.

[36] Wu S.-J., Pan S.-K., Wang H.-B., Wu J.-H. (2013). Preparation of chitooligosaccharides from cicada slough and their antibacterial activity. Int. J. Biolog. Macromol..

[37] Chandran R., Williams L., Hung A., Nowlin K., LaJeunesse D. (2016). SEM characterization of anatomical variation in chitin organization in insect and arthropod cuticles. Micron.

[38] Lavall R., Assis O., Campanafilho S. (2007). β-Chitin from the pens of Loligo sp.: Extraction and characterization. Bioresour. Technol..

[39] Vetter J. (2007). Chitin content of cultivated mushrooms Agaricus bisporus, Pleurotus ostreatus and Lentinula edodes. Food Chem..

[40] Ofenbeher-Miletić I., Stanimirović D., Stanimirović S. (1984). On determination of chitin content in mushrooms. Plant. Food Hum. Nutr..

[41] Abdelmalek B.E., Sila A., Haddar A., Bougatef A., Ayadi M.A. (2017). β-Chitin and chitosan from squid gladius: Biological activities of chitosan and its application as clarifying agent for apple juice. Int. J. Biolog. Macromol..

[42] Susana Cortizo M., Berghoff C.F., Alessandrini J.L. (2008). Characterization of chitin from Illex argentinus squid pen. Carbohydr. Polym..

[43] Ibitoye E.B., Lokman I.H., Hezmee M.N.M., Goh Y.M., Zuki A.B.Z., Jimoh A.A. (2018). Extraction and physicochemical characterization of chitin and chitosan isolated from house cricket. Biomed. Mater..

[44] Soon C.Y., Tee Y.B., Tan C.H., Rosnita A.T., Khalina A. (2018). Extraction and physicochemical characterization of chitin and chitosan from Zophobas morio larvae in varying sodium hydroxide concentration. Int. J. Biolog. Macromol..

[45] Kaya M., Baran T., Erdoğan S., Menteş A., Aşan Özüsağlam M., Çakmak Y.S. (2014). Physicochemical comparison of chitin and chitosan obtained from larvae and adult Colorado potato beetle (*Leptinotarsa decemlineata*). Mater. Sci. Eng. C.

[46] Tokatlı K., Demirdöven A. (2018). Optimization of chitin and chitosan production from shrimp wastes and characterization. J. Food Process. Preserv..

[47] Hossain M., Iqbal A. (2014). Production and characterization of chitosan from shrimp waste. J. Bangladesh. Agric. Univ..

[48] Hajji S., Younes I., Ghorbel-Bellaaj O., Hajji R., Rinaudo M., Nasri M., Jellouli K. (2014). Structural differences between chitin and chitosan extracted from three different marine sources. Int. J. Biolog. Macromol..

[49] Youn D.K., No H.K., Prinyawiwatkul W. (2009). Physicochemical and functional properties of chitosans prepared from shells of crabs harvested in three different years. Carbohydr. Polym..

[50] Tajik H., Moradi M., Rohani S., Erfani A., Jalali F. (2008). Preparation of Chitosan from Brine Shrimp (Artemia urmiana) Cyst Shells and Effects of Different Chemical Processing Sequences on the Physicochemical and Functional Properties of the Product. Molecules.

[51] Feás X., Vázquez-Tato M.P., Seijas J.A., Pratima G., Nikalje A., Fraga-López F. (2020). Extraction and Physicochemical Characterization of Chitin Derived from the Asian Hornet, Vespa velutina Lepeletier 1836 (Hym.: Vespidae). Molecules.

[52] Brugnerotto J., Lizardi J., Goycoolea F.M., Argüelles-Monal W., Desbrières J., Rinaudo M. (2001). An infrared investigation in relation with chitin and chitosan characterization. Polymer.

[53] Gbenebor O.P., Adeosun S.O., Adegbite A.A., Akinwande C. (2018). Organic and mineral acid demineralizations: Effects on *crangon* and *Liocarcinus vernalis*-sourced biopolymer yield and properties. J. Taibah Univ. Sci..

[54] Zhao D., Huang W.C., Guo N., Zhang S., Xue C., Mao X. (2019). Two-Step Separation of Chitin from Shrimp Shells Using Citric Acid and Deep Eutectic Solvents with the Assistance of Microwave. Polymers.

[55] Czechowska-Biskup R., Jarosińska D., Rokita B., Ulański P., Rosiak J.M. (2012). Determination of degree of deacetylation of chitosan-comparison of methods. Prog. Chem. Appl. Chitin Deriv..

[56] Roy J.C., Salaün F., Giraud S., Ferri A., Chen G., Guan J., Xu Z. (2017). Solubility of Chitin: Solvents, Solution Behaviors and Their Related Mechanisms. Solubility of Polysaccharides.

[57] Liu S., Sun J., Yu L., Zhang C., Bi J., Zhu F., Qu M., Jiang C., Yang Q. (2012). Extraction and Characterization of Chitin from the Beetle Holotrichia parallela Motschulsky. Molecules.

[58] Vázquez J., Ramos P., Mirón J., Valcarcel J., Sotelo C., Pérez-Martín R. (2017). Production of Chitin from Penaeus vannamei By-Products to Pilot Plant Scale Using a Combination of Enzymatic and Chemical Processes and Subsequent Optimization of the Chemical Production of Chitosan by Response Surface Methodology. Mar. Drug..

[59] Gbenebor O.P., Adeosun S.O., Lawal G.I., Jun S., Olaleye S.A. (2017). Acetylation, crystalline and morphological properties of structural polysaccharide from shrimp exoskeleton. Eng. Sci. Technol. Int. J..

[60] Ioelovich M. (2014). Crystallinity and Hydrophility of Chitin and Chitosan. J. Chem..

[61] Elieh-Ali-Komi D., Hamblin M.R. (2016). Chitin and Chitosan: Production and Application of Versatile Biomedical Nanomaterials. Int. J. Adv. Res. (Indore).

[62] Klapiszewski Ł., Bula K., Sobczak M., Jesionowski T. (2016). Influence of Processing Conditions on the Thermal Stability and Mechanical Properties of PP/Silica-Lignin Composites. Int. J. Polym. Sci..

[63] Fadli A., Drastinawati, Komalasari, Afriani Y., Maulana S., Huda F. (2017). Demineralization kinetics of chitin isolation from shrimp shell waste. Ces.

