# Supplementary material for: Extraction and Physicochemical Characterization of Chitin from Cicada orni Sloughs of the South-Eastern French Mediterranean Basin
Source: Molecules. 2020 May 29;25(11):2543. doi: 10.3390/molecules25112543 (PMC7321131; doi:10.3390/molecules25112543)
Supplement: Supplementary file 1 [file molecules-25-02543-s001.pdf]

Supplementary Materials

Extraction and physicochemical characterization of chitin from Cicada *orni* sloughs of south-eastern French Mediterranean basin

Aurelia Poerio[1], Chloé Petit[1], Jean-Philippe Jehl[1], Elmira Arab-Tehrany[2], João F. Mano[1,3], Franck Cleymand[1*]

[1] University of Lorraine, Jean Lamour Institute, UMR 7198 CNRS, 2 allée André Guinier-Campus Artem, BP 50840, F-54011 Nancy Cédex, France.
[2] University of Lorraine, Laboratoire Ingénierie des Biomolécules, TSA 40602, Vandoeuvre-lès-Nancy, F-54518, France.
[3] University of Aveiro, CICECO—Aveiro Institute of Materials, Department of Chemistry 3810-193 Aveiro, Portugal

* Correspondence: franck.cleymand@univ-lorraine.fr

Figure S1 shows how were traced the baselines for the calculation of the degree of acetylation, as porposed by Brugnerotto [49]. The values at 1420 and 1320 cm^{-1} were given by the length of the lines between the point of the peaks and the intersection with the baselines. There two values were then used in the Eq. (4). (See materials and methods).

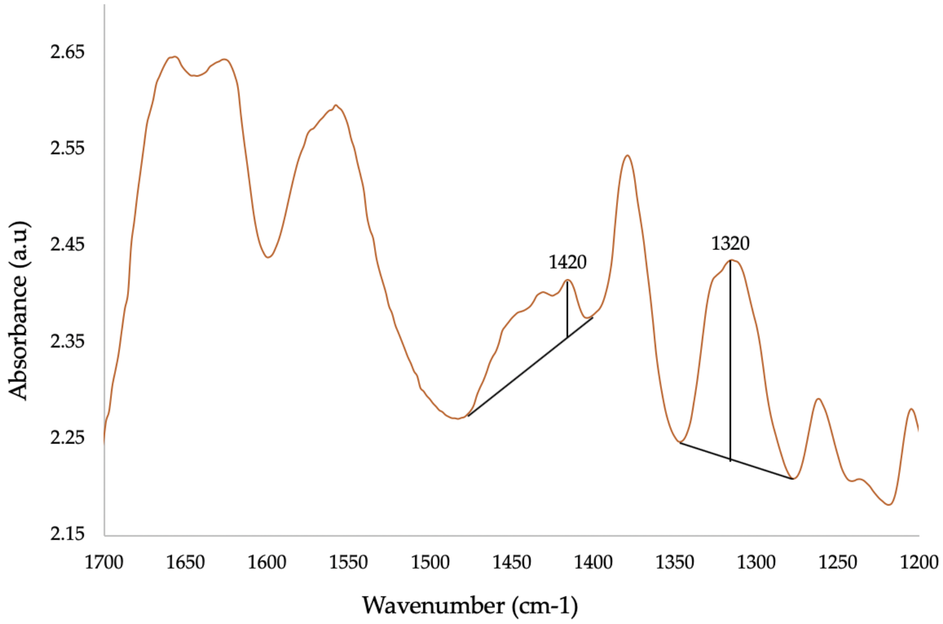

Figure S1. Representation of the baselines used to calculate the DA.

References

49. Brugnerotto, J.; Lizardi, J.; Goycoolea, F.M.; Argüelles-Monal, W.; Desbrières, J.; Rinaudo, M. An infrared investigation in relation with chitin and chitosan characterization. *Polymer* **2001**, *42*, 3569–3580.
